# Shaping the post-COVID-19 "New Normal" with Communication and Collaboration Platforms: state of the art communications for radiology, oncology, MDTs and beyond

**DOI:** 10.1259/bjro.20200038

**Published:** 2020-12-11

**Authors:** Robert James Johnson, Paula Wilson, Jon Hughes

**Affiliations:** 1 Worcestershire Acute Hospitals NHS Trust, England, United Kingdom; 2 Australian Telemedicine Clinic, Sydney, New South Wales, Australia

## Abstract

The COVID-19 pandemic has driven the use of digital communications to unprecedented levels across society whilst the NHS struggles with non-compatible IT systems that are often outdated and inhibit effective communication. MDTs use teleconferencing but the IT infrastructure does not permit clinicians to readily discuss cases and collaboratively review imaging outside of formal meetings if not on the same site and face-to-face. NHS radiology home reporting was not widely in place at the outbreak of the pandemic. Paper records persist further inhibiting remote working. Email has degraded the quality of written communication leading to suggestions of a ‘broken’ email culture. Despite NHS policy ambitions to address radiologist under capacity with increased networking and collaboration between providers the IT infrastructure has proven inadequate. Modern Communication and Collaboration Platforms have functionality that cuts across the non-compatible IT restrictions with screen sharing a key enabler. By engaging with these platforms radiologists and oncologists have a once-in-a-lifetime opportunity to shape the ‘new normal’ of delivery of healthcare with superior quality communication practices exceeding those in place at the outbreak of the pandemic.

## Introduction

The COVID-19 pandemic has been a great driver of change. Not only has it placed unprecedented demand on healthcare systems across the world but also presented new challenges on how we communicate and function in healthcare systems constrained by lockdown and social distancing.

Digital communication and teleconferencing are at an all-time high. The World Economic Forum reported an increase of one platform, ‘*Zoom*’, from 10 million daily meetings in December 2019 to 200 million in March 2020.^1^

Consequently, this raises the issue of public expectations of the NHS’s readiness to respond and move to remote working practices. Government and the media are referring to a ‘new normal’. With existing but as yet underutilised digital communication and collaboration platforms (CCPs), there is a great opportunity for clinicians to shape this ‘new normal’, not only in response to the COVID-19 pandemic but for the future benefit of patient care across the NHS.

## Background

Radiology and oncology were at the forefront of adopting IT developments. Picture Archiving and Communication Systems (PACS) and videoconferencing for Multidisciplinary Team meetings (MDTs) are well established. However, these are often not seamlessly integrated, with many clinicians using multiple IT systems to treat one patient.^2^ Whilst single sign-on solutions to multiple applications such as email, order communications systems and a CCP could avoid adding to the ‘password burden’, widespread access to teleconferencing for *ad hoc* case discussion between colleagues is not established. Many NHS trusts’ teleconferencing solutions are too complex and remain in locked meeting rooms inaccessible outside of dedicated MDT meetings. MDTs perform an essential role, yet there is a need for more *ad hoc* collaborative case discussions that are currently inhibited by restricted access to teleconferencing.

Such variation of IT systems is further compounded by out-of-date technology and mixed use of paper and digital medical records. Until recently, FAX machines persisted in the NHS and their removal by the end of April 2020 had to be mandated by the Health and Social Care Secretary.^3^ In July 2019, over 2000 NHS computers were running Windows XP^4^; 94% of NHS hospitals still use handwritten notes according to a 2018 parliamentary report.^5^ Paper requests to radiology departments persist and the NHS Benchmarking Report 2018 revealed that 25% of radiology requests were outside of digital order communications systems. IT systems between different institutions do not integrate seamlessly and can inhibit effective case discussion.

For radiology, NHS Benchmarking revealed that only 18% of the consultant radiologist workforce had access to home reporting capability in 2018. At the outbreak of the COVID-19 pandemic, the RCR decided to relax their guidance standards for display devices for use in home reporting by permitting the use of off-the-shelf computer displays in an attempt to rapidly increase UK radiologist home working capacity.^6^

Increased radiology networking has been advised by NHS Improvement and their 2019 white paper: *Transforming imaging services in England: a national strategy for imaging* makes multiple recommendations for alignment between providers and increased clinical networking.^7^ When such networks are created between providers another layer of complexity can be introduced. Rarely are PACS networks connected and existing methods for transferring images, such as the Image Exchange Portal are cumbersome and not instantly available at the time of patient care.

This lack of harmonisation acts as an impediment to effective clinical communication and efficient use of clinician’s time. Until IT networks are fully integrated, realising the benefits of optimised use of resources and seamless sharing of large data sets (whilst enabling new technologies such as artificial intelligence and machine learning), CCPs can facilitate interaction between existing non-compliant systems.

## Increasing quantity and quality of communication

Clinician and radiologist discussion leads to better patient outcomes.^8^ Howlett et al state *There are significant reductions in major discrepancy and patients coming to harm with increased seniority and the reporting radiologist onsite*.^9^ The assumption has been that remote working in radiology is an impediment to good communication. Howlett et al acknowledge the UK radiology reporting capacity shortfall and insufficient numbers of subspecialty radiologists available on every hospital site.

## Collaborative communication platforms

The technology has progressed from instant messaging programs into effective CCPs alongside smartphone advances. Most provide group messaging, voice and video calling, file sharing and screen sharing. These CCPs are ideally suited to modern healthcare delivery.

## Screen sharing

Formal screen sharing technology is used in MDTs and is a feature of many CCPs. There are shortcomings of network bandwidth and screen refresh rates although the functionality continues to improve. Nevertheless, screen sharing cuts across the limitations of non-integrated PACS and other IT systems. The key enabler is that it is the monitor display output that is shared regardless of the application or program in use. This enables case-based discussion between clinicians who can jointly review clinical data such as radiology on PACS or medical photographs, endoscopic and surgical images, pathology etc. Screen sharing is live allowing image manipulation and pointing out the findings as we are familiar with in MDTs but in more widely accessible locations, *i.e*. on a PC or a mobile device. There are many benefits to *ad hoc* case discussions outside of formal MDTs (which we do not propose are replaced). A surgeon who reviews the imaging in theatre may benefit from the input of a radiologist part way through an operation and a CCP would enable this regardless of the locality of the radiologist. Most point-of-care locations already have IT hardware capable of supporting CCP use. All clinicians must maintain the established rules of confidentiality and diligence to correct patient demographics regardless of the hardware used. We do not propose that CCPs are used for primary radiology reporting just as established MDTs are not the forum for radiology reporting.

## Messaging

Most CCPs have a messenger function. In group-enabled forums, if a written communication is sent to a group and a member of the group replies, this can be seen by other group members and no further action need be taken. Before messaging has become established, the use of email in the NHS led to a blurring of the boundaries of what constitutes high quality written communication. Increased time spent differentiating the genuine clinical correspondence from the proliferation of junk can cause anxiety with many clinicians resorting to monitoring their email outside of work hours with a negative impact on wellbeing.^10^ Many emails would be better sent as messages. If email is considered to represent formal letters the differentiation should become clear.

## Teams not firms

As these CCPs have become increasingly available on our own mobile devices, they have already proven a temptation to many clinicians, particularly those in changing teams and shift working where teams can be rapidly set up for clinical communication. Clinicians must comply with General Data Protection Regulations (GDPR) and 2018 NHS Guidance on the topic makes recommendations on the available programs/applications and the Information Commissioner’s guidance on using personal smartphones.^11^

## NSH.net adopts MS teams

Microsoft’s *Teams* CCP has recently been adopted by NHS.net.^12^ *Teams* includes all the functionality described above. Collaboration can take place across a wide variety of clinical settings and geographies cutting across the constraints of IT variations between providers and rapidly changing clinical teams.

We argue that it is the quality of the communication and not the site it takes place on that is key. The ‘silo’ based model of radiologists only reporting for an individual hospital or trust is needlessly restrictive when the technology exists to connect subspecialty experience with areas of need. Moreover, networks linked with CCPs can facilitate high quality communication. The principle hurdle is overcoming entrenched resistance to change. The ‘new normal’ will require a cultural shift but the COVID-19 driver is forcing greater levels of engagement with these platforms. It has challenged the assumption that communication in person is the gold-standard. The CCPs available should not be viewed as temporary workarounds as they facilitate far more than historical communication methods.

If NHS radiology is to align its workforce to achieve the ambitions set out in the *Transforming Imaging Services* white paper,^7^ then CCPs such as *Teams* could be an effective tool to allow collaboration and overcome the hurdles of variations in IT systems across providers. Some teleradiology companies are already using this technology and are achieving collaboration across team members located across the globe connected to the NHS.

## Concluding comments

Governments and wider society are debating and adapting to the ‘new normal’. The COVID-19 pandemic has forced new ways of working and communicating on us all. The NHS is in a unique position to build radiology and oncology networks within its infrastructure. By making *Teams* available through NHS.net, there are many opportunities for more efficient team working and collaboration for radiologists, oncologists and clinicians. In the post-COVID-19 era, communication with colleagues will less likely be face-to-face. We the authors encourage the uptake of CCPs such as *Teams* wholeheartedly with a transition away from a broken email culture, non-compatible IT solutions and silo working in a step towards a future of superior medical communications where collaboration is readily available across networks matching clinical need.
